# Strokectomy for malignant middle cerebral artery infarction: experience and meta-analysis of current evidence

**DOI:** 10.1007/s00415-020-10358-9

**Published:** 2020-12-19

**Authors:** Saad Moughal, Sarah Trippier, Alaa AL-Mousa, Atticus H. Hainsworth, Anthony C. Pereira, Pawanjit S Minhas, Anan Shtaya

**Affiliations:** 1 Neurosciences Research Centre, Molecular and Clinical Sciences Research Institute, St George’s, University of London, London, SW17 0RE UK; 2 Neurology Department, St George’s University Hospitals NHS Foundation Trust, London, UK; 3 Department of Surgery, Faculty of Medicine, The Hashemite University, Zarqa, Jordan; 4 Atkinson Morley Neurosurgery Centre, St George’s University Hospitals NHS Foundation Trust, London, UK

**Keywords:** Malignant MCA infarction, Decompressive craniectomy, Strokectomy

## Abstract

Strokectomy means surgical excision of infarcted brain tissue post-stroke with preservation of skull integrity, distinguishing it from decompressive hemicraniectomy. Both can mitigate malignant middle cerebral artery (MCA) syndrome but evidence regarding strokectomy is sparse. Here, we report our data and meta-analysis of strokectomy compared to hemicraniectomy for malignant MCA infarction. All malignant MCA stroke cases requiring surgical intervention in a large tertiary centre (January 2012–December 2017, *N* = 24) were analysed for craniotomy diameter, complications, length of follow-up and outcome measured using the modified Rankin score (mRS). Good outcome was defined as mRS 0–3 at 12 months. In a meta-analysis, outcome from strokectomy (pooled from our cohort and published strokectomy studies) was compared with hemicraniectomy (our cohort pooled with published DECIMAL, DESTINY and HAMLET clinical trial data). In our series (N = 24, 12/12 F/M; mean age: 45.83 ± 8.91, range 29–63 years), 4 patients underwent strokectomy (SC) and 20 hemicraniectomy (HC). Among SC patients, craniotomy diameter was smaller, relative to HC patients (86 ± 13.10 mm, 120 ± 4.10 mm, respectively; *p* = 0.003), complications were less common (25%, 55%) and poor outcomes were less common (25%, 70%). In the pooled data (*N* = 41 SC, 71 HC), strokectomy tended towards good outcome more than hemicraniectomy (OR 2.2, 95% CI 0.99–4.7; *p* = 0.051). In conclusion, strokectomy may be non-inferior, lower risk and cost saving relative to hemicraniectomy sufficiently to be worthy of further investigation and maybe a randomised trial.

## Introduction

Malignant middle cerebral artery (MCA) infarction is a life-threatening medical emergency that carries a poor prognosis with mortality of up to 80% in untreated patients [10, 15]. In malignant MCA syndrome expanding oedema causes mass effect, a substantial rise in intracranial pressure (ICP) and reduction of cerebral blood flow (CBF) [11]. Malignant MCA syndrome can cause further infarction in other vascular territories especially the anterior cerebral artery [15]. Medical treatment alone to reduce the raised intracranial pressure is not effective [10, 14, 15].

Decompressive hemicraniectomy (hemicraniectomy) significantly reduces mortality and improves functional outcome following malignant MCA infarction [4]. The initial three randomised controlled trials (DECIMAL [40], HAMLET [13] and DESTINY [18]) considered individually or with pooled patient data analysis demonstrated that hemicraniectomy was superior to medical management alone in both survival and functional outcome measured using the modified Rankin score (mRS) where good outcome was defined as mRS ≤ 3 [13, 18, 39, 40]. The objective with hemicraniectomy is to avoid secondary brain damage from elevated ICP and herniation. Hemicraniectomy survivors must undergo a second surgical procedure to close the skull defect (cranioplasty). This procedure carries risks from evolving brain injury while awaiting cranioplasty and also from potential complications of the procedure itself, which include infection and air embolism [33]. Hemicraniectomy can also cause a syndrome of the trephined [1] or hydrocephalus [27]. Therefore, hemicraniectomy while improving survival and outcome also comes with significant risk and additional cost.

Partial resection of infarcted frontal and/or temporal lobe with preservation of skull integrity, termed “strokectomy”, has been suggested either as an adjuvant or surgical alternative to hemicraniectomy to effect decompression [23, 35, 38]. Surgical decompression (including strokectomy) for cerebellar infarction is well established and associated with improved outcome [16, 19, 29, 37]. Whether supratentorial or infratentorial, the rationale of strokectomy is to resect just enough infarcted brain tissue to alleviate the deleterious effects of progressive cerebral oedema, allowing the bone flap to be replaced immediately. This precludes the need for cranioplasty and its associated complications [9, 33]. In addition, removal of the anterior temporal lobe may rapidly relieve brainstem compression. The main risk is that resection of infarcted brain may not be sufficient to contain the oedema, and the decompression could be overwhelmed by further swelling.

In this study, we present a small case series of patients with malignant MCA infarction who underwent either strokectomy or hemicraniectomy as a primary procedure. We also performed a meta-analysis of our data combined with published outcome data from other strokectomy reports and three hemicraniectomy randomised trials (DECIMAL, DESTINY, HAMLET).

## Methods

This retrospective cohort study was registered as an audit with our institutional approval (AUDI000525). A database was created for all malignant MCA stroke cases requiring surgical intervention during the period from January 2012 to December 2017 inclusive. Data were analysed for patients’ age, sex, comorbidities, neurological status (GCS), side of the stroke, size of craniotomy, complications, length of follow-up and outcome measured using the modified Rankin score (mRS). Computed tomography (CT) scan of the head was the imaging used for diagnosis of MCA stroke. Patients received medical therapy that included oxygen, diuretics, mannitol and/or hypertonic saline infusion as appropriate on the ward but still deteriorated in terms of drop in conscious level (stop obeying commands). Surgery was indicated subject to clinical and imaging evaluation by experienced neurosurgeons. Midline shift of > 5 mm; involvement of 2/3 of the MCA territory; signs of trans-tentorial herniation or progressive worsening of neurological status indicated emergency decompression. The stroke and neurosurgery teams discussed each case thoroughly before proceeding to surgery. The surgical procedure including the technique was discussed with the patient (if appropriate) or next of kin before proceeding in the patient’s best interests.

### Surgical procedure

**Fig. 1:**
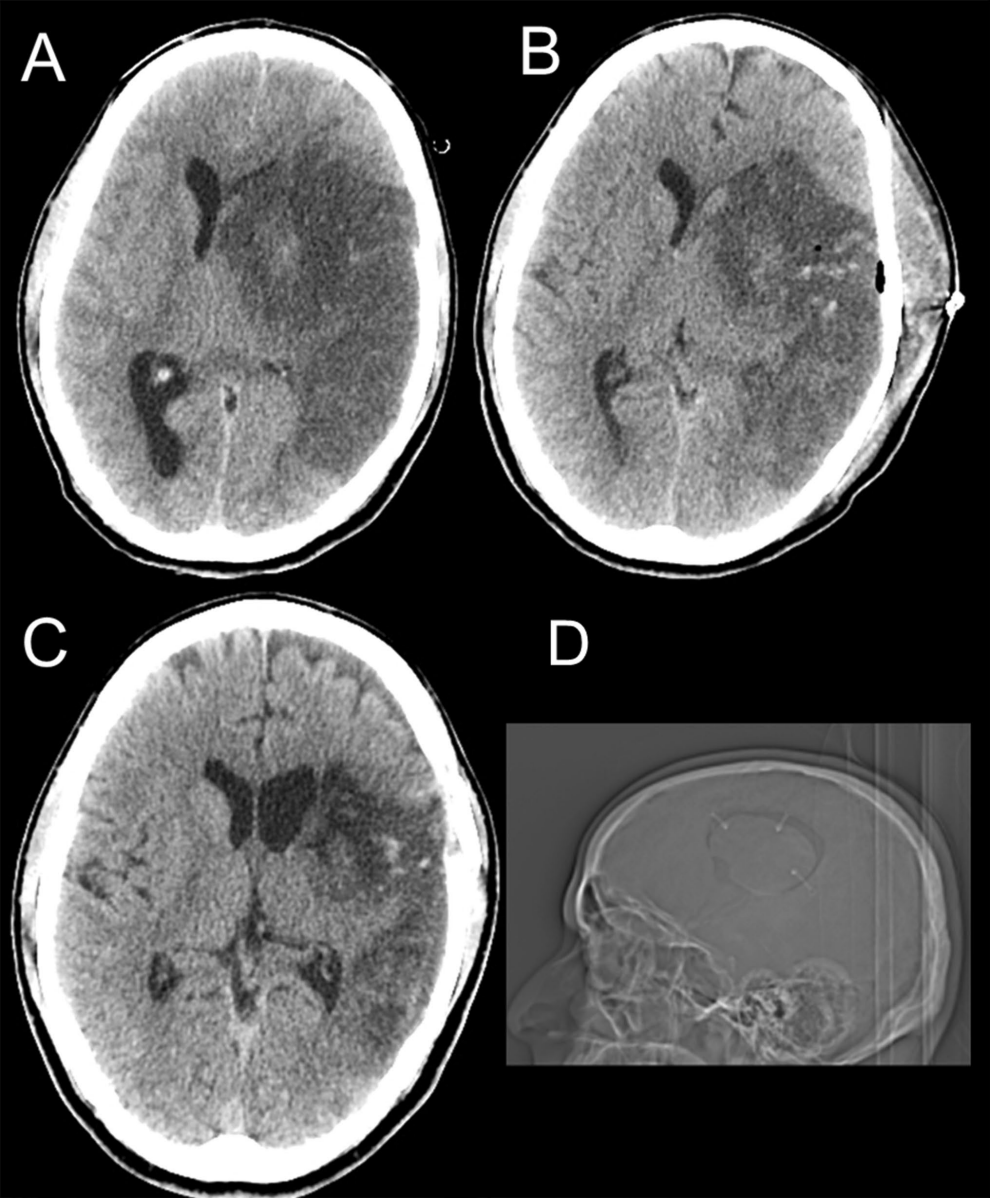
A 36-year-old male with malignant L MCA stroke. **a** Preoperative axial CT scan demonstrating severe midline shift. **b** 48-h postoperative axial CT scan with debulking of ischemic tissue from the left temporal lobe, there is evident midline shift despite clinical improvement in neurological status. **c** An axial CT head of the same patient at 1 month following surgery. **d** This is a skull scout image, showing the craniotomy size, the free riding mini-plates attached to the craniotomy flap only and that the bone did not sink into the cranial vault 3 years post-surgery. This patient recovered to mRS 2

In our institution, the main surgical procedure for malignant MCA stroke is hemicraniectomy. However, we also do strokectomy for cerebellar infarcts and Mr P Minhas introduced strokectomy as a potential one-off definitive procedure for malignant MCA infarction. Whenever Mr P Minhas receives a referral of malignant MCA infarction, he reviews all CT scans of the patient and considers whether strokectomy maybe a simpler alternative. It is then performed by his surgical team under close supervision. All patients with malignant middle cerebral artery syndrome who were referred during Mr Minhas’s on call and required surgical treatment went for strokectomy. After the consent procedure, emergency anaesthetic preparation included transfusion of 1–2 pools of platelets to patients on high dose or dual antiplatelet therapy. At least one pool of platelets was given prior to surgery. Mannitol and hypertonic saline were considered on a case-by-case basis. Surgically, a curved temporal incision was made anterior to the tragus followed by a small standard craniotomy tailored to the ischaemic brain tissue area. The dura was opened in a C-shape and reflected. The ischaemic tissue was identified as non-viable blanched brain tissue, soft, grey, swollen with no evidence of perfusion. Subpial resection was performed with assistance of suction and bipolar forceps. Infarcted brain undergoes liquefactive necrosis and, hence, is possible to use gentle suction and irrigation to resect infarcted tissue without disturbing viable brain. Sufficient ischaemic brain tissue was resected to optimise a manageable frontal and/or temporal lobe and be able to replace the bone flap. The dura was left open and the bone flap was replaced free riding with attached plates to maintain convexity integrity. Patients were admitted to intensive care following surgery. CT head was performed within 48 h of surgery or as clinically needed. Antithrombotic therapy was re-started after reviewing the CT as necessary. Figure 1 demonstrates malignant left MCA and PCA territory infarction in a 36-year-old male who deteriorated following admission and had surgery at 46 h from the onset of stroke.

**Fig. 2:**
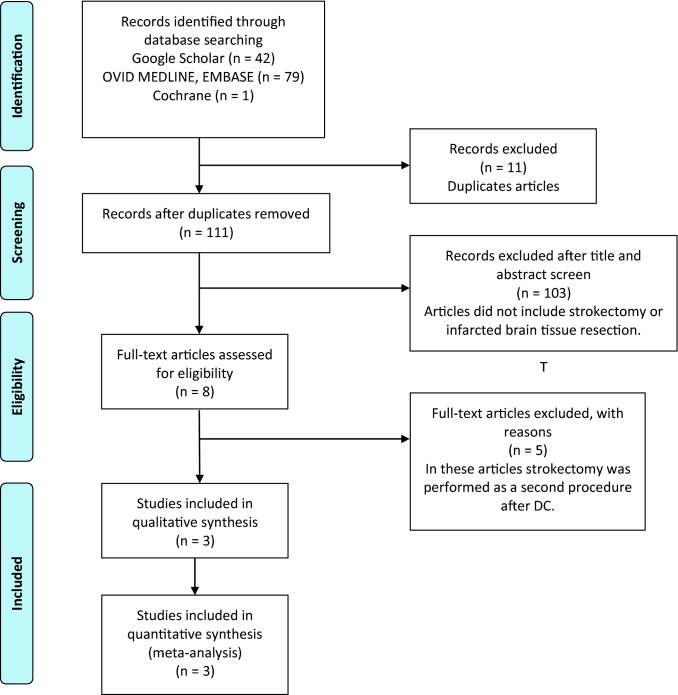
PRISMA flow diagram of Google scholar, PubMed, Ovid, Medline, Embase and Cochrane until December 2019

The pre-operative plan was to perform strokectomy and hemicraniectomy was not an option. We are not aware of any selection bias as strokectomy is the strategy of Mr Minhas practise, and all patients referred during his on call had this procedure (Fig. 2).

### Outcome

Outcome was measured using the mRS by independent clinicians immediately preoperatively and 12 months post-operatively. Patients, their next of kin or carers were contacted for a 12-month mRS if they did not attend in person. Outcome was dichotomised into ‘good’ if mRS ≤ 3 or ‘poor’ if mRS ≥ 4.

### Search strategy for systematic review

An extensive literature search was performed including PubMed, Google Scholar, OVID, EMBASE and the Cochrane Central Register of Controlled Trials (CENTRAL). Limits were placed on all articles to those published between 1990 and 2019 inclusive and written in English only. Search terms were charted to subject headings and combined using Boolean operations. The following keywords were used for search: “natural history”, “malignant infarction”, “supratentorial infarction”, “risk factors”, “survival rates”, “decompressive craniectomy”, “strokectomy”, “infarcted tissue removal”, “medical therapy”. Abstracts of papers found in the literature search were scrutinised independently by two authors (SM and AS) to assess suitability for inclusion. Reference lists from the papers identified in the literature search were manually searched to ascertain other articles suitable for inclusion. Inclusion criteria were: any article that described malignant MCA infarction patients who underwent infarcted brain tissue resection either as a primary procedure or secondary surgery following hemicraniectomy. Primary procedure was when strokectomy was the intended operation, while secondary procedure was when a resection of infarcted brain tissue was performed in addition to hemicraniectomy in the same setting or at a later operation to reduce ICP. In the meta-analysis, we included all articles that strokectomy was performed as primary surgical procedure in the treatment of malignant MCA infarction. Those with no outcome or when strokectomy was performed as secondary procedure were excluded.

The systematic review and meta-analysis were performed following PRISMA guidelines.

### Statistical analysis

For our cohort data analysis: continuous numeric variables are summarised as mean and standard error of the mean. The differences in the numeric variables of the two groups were evaluated with a Mann–Whitney *U* test for non-parametric statistical analysis. Chi-square with Fisher's exact test was employed to compare categorical data between the groups. Statistical significance was considered if *p* < 0.05. Statistical analyses were performed using GraphPad Prism version 8.02 for Windows 10, GraphPad Software, La Jolla, CA, USA.

For the systematic review, strokectomy data were analysed using patient outcomes from our data pooled with data from three strokectomy studies [20, 22, 38] and compared to pooled hemicraniectomy data taken from our data and the hemicraniectomy studies “DECIMAL, DESTINY and HAMLET” [39]. In total, the pooled dataset consisted of 41 patients in the strokectomy group and 71 patients in the hemicraniectomy group. A good outcome was defined as mRS 0–3 with poor outcome separated into mRS 4 and 5 or death (mRS 6) at 12 months. Absolute risk reduction (ARR), odds ratio (OR) and 95% confidence intervals were calculated for our data and pooled data where possible. Chi-squared was used for the pooled dataset and Fisher’s Exact test for our dataset to compare the two interventions.

## Results

Patients’ characteristics from the present cohort are summarised in Table 1. Four hemicraniectomy patients had ICA, ACA and PCA territory infarction, while two of the strokectomy patients had additional ACA and ICA territory infarcts. No patients presented with pupillary abnormality or cardiovascular instability before surgery. There was no significant difference between the two groups in any of the listed clinical demographics, pre-surgery GCS or mRS. In the hemicraniectomy group, none of the patients had secondary strokectomy (or temporal lobectomy). Likewise, none of the strokectomy group patients had secondary hemicraniectomy.

**Table 1:** Baseline demographics and clinical data of hemicraniectomy and strokectomy of local department cohorts

	Hemicraniectomy	Strokectomy	*p* value
*N*	20	4	–
Male	9	3	0.59
Female	11	1	
Age	44.7 ± 1.8	51.5 ± 5.97	0.19
Side	7 left, 13 right	2 left, 2 right	0.61
Treatment with rtPA	12	3	0.30
Thrombectomy	3	0	–
Stroke type	13	3	–
Atherothrombotic	6	0	–
Cardio-embolic	Carotid dissection (*n* = 1)	Carotid dissection (*n* = 1)	–
Other			
Infarction territory	20 MCA, additionally 1 ICA, 2 ACA and 1 PCA	4 MCA, additionally 1 ICA and 1 ACA	–
NIHSS (on admission)	15 (range 6–24)	21.5 (range 17–25)	0.06
NIHSS (pre-operative)	21 (range 10–35)	28 (range 23–31)	0.04*
GCS (pre-operative)	9 (range 6–13)	8 (range 3–12)	0.30
mRS (on admission)	5 (range 3–5)	5 (range 4–5)	0.87
mRS (at follow-up)	4 (range 1–6)	3 (range 2–4)	0.08**
Time between stroke and surgery	33.0 ± 2.8 h (range 12–60)	41.3 ± 5.8 h (range 24–48)	0.30

rtPA: recombinant tissue plasminogen activator. mRS: Modified Rankin score. NIHSS: National Institutes of Health Stroke Scale. Age and time “stroke-to-surgery” are expressed as mean ± standard error of the mean. NIHSS, GCS and mRS are expressed as median. *p < 0.05 is significant. **Mann–Witney test was used in this analysis

### Craniotomy diameter in hemicraniectomy versus strokectomy

The average craniotomy diameter was significantly smaller in the strokectomy compared to the hemicraniectomy cohorts (85 ± 4.1 mm vs. 120 ± 13.1 mm; *p* = 0.003).

### Patients’ outcome

**Fig. 3:**
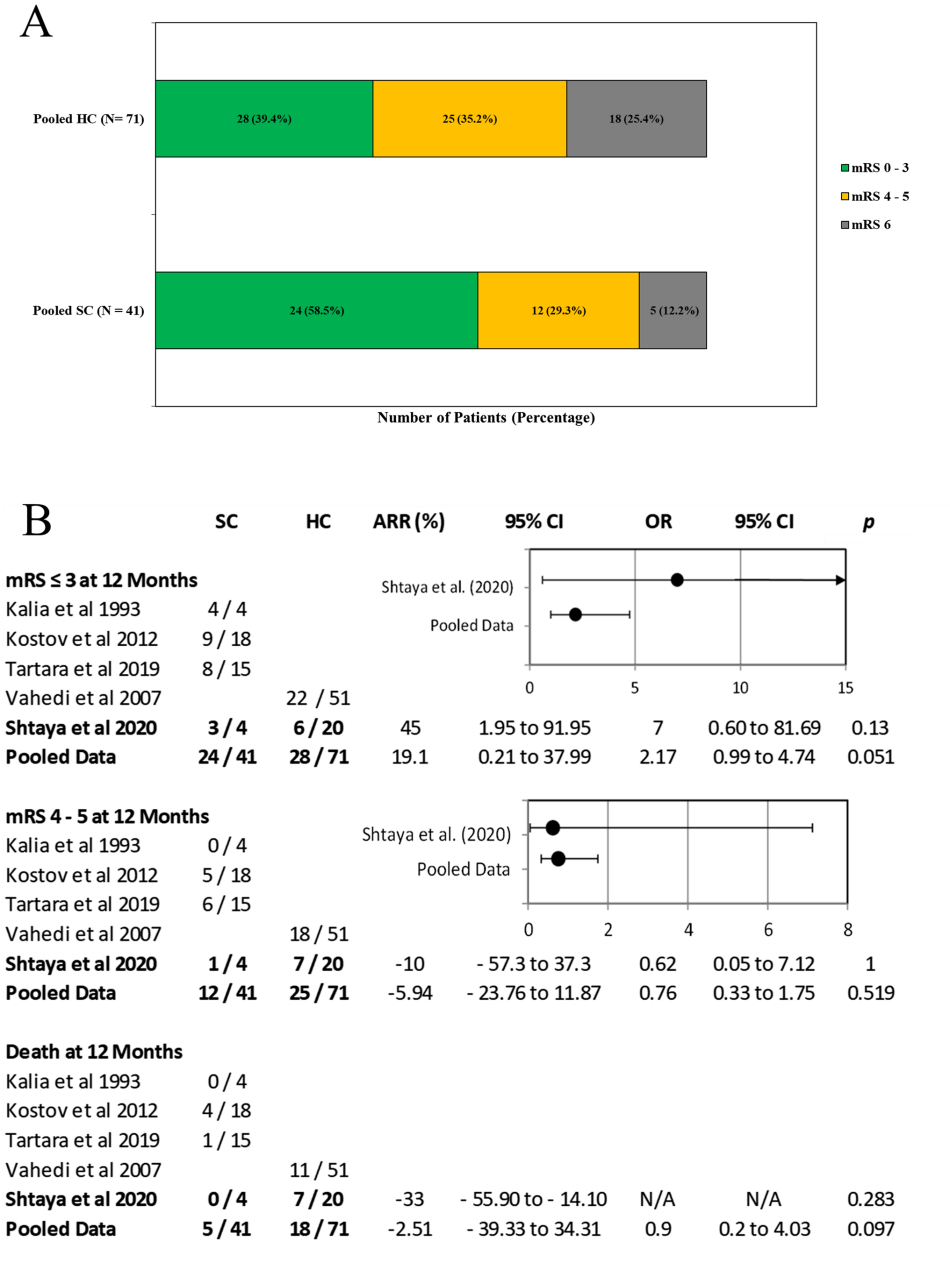
Comparison between strokectomy (SC) and hemicraniectomy (HC), our data and pooled meta-analysis data. **a** Distributions of the scores on the mRS and death after 12 months for patients treated with SC or HC. **b** Absolute risk reduction and odd ratio for good and poor outcomes at 12 months

The average in-person clinic follow-up for both groups was 14 months (range 3–36). Those who did not have a 12-month clinic review were contacted and their mRS was recorded. Median mRS at 12 months was 3 (range 2–4) for strokectomy and 4 (range 1–6) for hemicraniectomy (*p* = 0.08). Mortality at one year was zero (0/4) in the strokectomy group and 35% (7/20) in the hemicraniectomy group. A good outcome (mRS ≤ 3) was observed in 75% (3/4) and 30% (6/20) of patients in the strokectomy and hemicraniectomy cohorts, respectively (Fig. 3) (Tables 2 and 3).

**Table 2:** Studies that included malignant MCA infarction patients who underwent strokectomy or resection of infarcted brain tissue either as a primary surgery or as an adjuvant secondary procedure

Article	Primary or secondary strokectomy	Number of patients with Strokectomy	Outcome available (Y/N)	Included in analysis Y/N
Kalia et al. (1993)	Primary	4	Y	Y
Cho et al. (2003)	Secondary	13	Y	N
Kostov et al. (2012)	Primary	18	Y	Y
Lee et al. (2013)	Secondary	26	Y	N
Merenda et al. (2015)	Secondary	3	Y	N
Kürten et al. (2018)	Secondary	20	Y	N
Schwake et al. (2019)	Secondary	12	Y	N
Tartara et al. 2019	Primary	15	Y	Y

Y = Yes, N = No

**Table 3:** Studies that included malignant MCA infarction patients who underwent strokectomy or resection of infarcted brain tissue as a primary surgery and included in the meta-analysis

	Number of patients (%)	Mean age	M/F (%)	R/L	Time from stroke to surgery (h)	NIHSS on admission	NIHSS pre-operative	Good outcome	Poor outcome	
								mRS ≤ 3 (%)	mRS 4–5 (%)	mRS 6 (Mortality) (%)
Kalia et al. (1993)	4 (10)	34.25 ± 7.9 Y (range 13–47)	2 M (5) 2 F (5)	2 R 2 L	NA	NA	NA	4 (10)	0	0
Kostov et al. (2012)	18 (44)	51 ± 13 Y (range N/A)	11 M (27) 7 F (17)	13 R 5 L	46 ± 41 (median 28)	NA	NA	9 (22)	5 (12)	4 (10)
Tartara et al. (2019)	15 (36)	61.7 ± 9.3 Y (range 38–72)	9 M (22) 6 F (15)	10 R 5 L	52.7 ± 19.3 (range 24–96)	19.7 ± 2.3 (range 18–23)	26.2 ± 1.3 (range 24–28)	8 (20)	6 (15)	1 (2)
Shtaya et al. (2020) (current study)	4 (10)	51.5 ± 5.97 Y (range 36–63)	3 M (7) 1 F (2)	2 R 2 L	41.3 ± 5.8 (range 24–48)	21.5 (range 17–25)	28 (range 23–31)	3 (7)	1 (2)	0

M: Male, F: Female, R: Right, L: Left, Y: year, mRS: Modified Rankin score, h: hour, NIHSS: National Institutes of Health Stroke Scale, NA: not available, % percentage is displayed as part of the overall number (41 cases included)

### Complications

In the strokectomy group, 1 patient (25%) developed pneumocephalus and then recovered with outcome mRS = 4. In the hemicraniectomy group, four patients (20%) developed seizures (2 shortly after the surgery and two following cranioplasty). All 4 had poor neurological status on admission and poor outcomes. A further four patients (20%) in this group developed pneumonia which was treated successfully with antibiotics. One patient developed hydrocephalus and another patient developed atrial fibrillation.

Six patients out of the 13 who survived in the hemicraniectomy group underwent cranioplasty where two developed seizures. The remaining seven patients required nursing home care and were fully dependent; they were considered unsuitable for cranioplasty.

### Systematic review and meta-analysis

Among 122 retrieved studies, 114 were excluded because of duplicates or because titles and abstracts did not meet the inclusion criteria (Fig. 2). In the remaining eight, full texts were assessed for eligibility and 5 were excluded because they did not include strokectomy as a primary procedure for the treatment of malignant MCA infarction [5, 23–25, 35]. We included three studies [20, 22, 38] with a total of 37 patients. Overall, the SC dataset comprised four studies (including ours) for outcome analysis (pooled *N* = 41) [20, 22, 38]. For the HC analysis, we pooled data from DECIMAL [40], DESTINY [18] and HAMLET [39]” studies with our data (pooled N = 71 patients). Both in our data and in the pooled data, there was a trend for better outcomes for strokectomy than for hemicraniectomy (for mRS ≤ 3, our data: OR 7.0, 95% CI 0.6—81, pooled data: OR 2.2, 95% CI 0.99–4.7; Fig. 3a and b).

## Discussion

The main finding in our report is that from the available evidence, strokectomy may have a role in the surgical management of supratentorial malignant stroke syndromes. It may provide a simpler operative procedure with potentially fewer complications and avoid the cost and complications of a secondary cranioplasty procedure. It appears to be of sufficient promise for further investigation and maybe a trial versus decompressive hemicraniectomy to better evaluate the risks, benefits and comparative merits of the two surgical strategies.

Hemicraniectomy is superior to standard medical therapy (without surgery) and greatly improves the survival and functional outcome of patients with malignant MCA infarction. [7, 39] However, hemicraniectomy as a surgical procedure has inherent complications that include infection, haemorrhage, CSF disturbance and seizures. [2, 3, 6, 19, 26, 34] Hydrocephalus is reported prior to and following hemicraniectomy, and is associated with significant morbidity. [32, 34, 36] Syndrome of the trephined is another complication that has been described and arises from the loss of skull integrity from leaving the bone flap out. It is characterised by cognitive and neurological disturbance but normally improves following cranioplasty. [1] Hemicraniectomy usually entails a second operation, cranioplasty, performed several months later. Cranioplasty brings additional costs, in the range of $25,000–29,000 [8] and there is controversy surrounding timing, bone flap preservation and complications that include infection, seizures, hydrocephalus, further neurological damage and death. [6, 9, 17, 30, 33] There is also a theoretical concern that herniated brain after hemicraniectomy has impaired venous return which leads to further infarction [12, 28, 41] which can be avoided in strokectomy. Our analyses show a clear trend towards improved outcome in the strokectomy group when compared to hemicraniectomy.

Strokectomy has been widely adopted in the treatment of cerebellar ischaemic infarcts. [21, 29, 31, 37] Some elements of strokectomy are already used such as supratentorial resection of infarcted brain tissue including the temporal pole as an adjuvant procedure following hemicraniectomy either to control ICP intraoperatively or when there is a refractory rise in ICP. [20, 23–25, 35] Strokectomy as a primary surgical procedure where the bone flap is replaced has been described in small series. [20, 22, 38] Our technique is slightly different and craniotomies in our strokectomy group were significantly smaller when compared to those who underwent hemicraniectomy. Likewise, Tartara et al. independently reported smaller craniotomies in strokectomy cases. [38] In our cohort, we had 75% good outcome and no mortality. Complications were minimal after the procedure and none of the patients had post-surgery haemorrhage. Haemostasis was satisfactory during surgery with no bleeding encountered in the subpial resection of the dead tissue and the surgery was straightforward. Although we were prepared to do secondary craniectomy for the strokectomy patients, none in our cohort needed that. Our cohort is small and data should interpreted with caution; larger studies are needed.

Our literature review identified that supratentorial strokectomy procedure was being carried out in some centres either as a primary operation or a secondary procedure for a refractory rise in ICP. [5, 20, 22–25, 35, 38] Our local data concur with the published literature which suggests strokectomy is safe, and may be not be inferior to hemicraniectomy, in the management of malignant MCA infarction. Pooling the outcome results of four studies that included strokectomy as a primary procedure, we report survival of 88% and good outcome in 59%. There may be a trend towards significantly improved outcome. The hemicraniectomy data from Vahedi et al. included patients with NIHSS > 15, those between 18 and 60 years old and operation performed within 45 h of stroke; these may not accurately match the data from the strokectomy group. This comes as one of the limitations of this retrospective analysis and should be taken into consideration in future studies.

## Limitations

Our retrospective, local study of strokectomy patients was too small for definitive conclusions to be drawn. Such a small series may also be prone to selection bias, although, we are not aware of a systematic bias in selecting these patients. Given that the metanalysis was not a randomised (nor systematically selected) sample, it is likely the baseline characteristics in the two groups may not be balanced. However, while our study was small, the data are robust to be considered as hypothesis generating.

## Conclusion

Our study suggests that strokectomy can be used safely to manage malignant middle cerebral artery syndrome and has some prospective advantages over hemicraniectomy, such as the avoidance of bone reconstruction and its sequelae. These findings support further evaluation of the safety and efficacy of this approach and potentially a clinical trial.

## Data Availability

Researchers can apply for access to anonymized data from the present study for well-defined research questions that are in line with the overall research agenda for the cohort. Please contact the corresponding author.
